# HIF-1α promotes SARS-CoV-2 infection and aggravates inflammatory responses to COVID-19

**DOI:** 10.1038/s41392-021-00726-w

**Published:** 2021-08-18

**Authors:** Mingfu Tian, Weiyong Liu, Xiang Li, Peiyi Zhao, Muhammad Adnan Shereen, Chengliang Zhu, Shanyu Huang, Siyu Liu, Xiao Yu, Miaomiao Yue, Pan Pan, Wenbiao Wang, Yongkui Li, Xulin Chen, Kailang Wu, Zhen Luo, Qiwei Zhang, Jianguo Wu

**Affiliations:** 1 The First Affiliated Hospital, Jinan University, Guangzhou, China; 2 Guangdong Provincial Key Laboratory of Virology, Institute of Medical Microbiology, Jinan University, Guangzhou, China; 3 Department of Clinical Laboratory, Tongji Hospital, Huazhong University of Science and Technology, Wuhan, China; 4 Hubei Provincial Center for Disease Control and Prevention, Wuhan, China; 5 State Key Laboratory of Virology, College of Life Sciences, Wuhan University, Wuhan, China; 6 Department of Clinical Laboratory, Renmin Hospital, Wuhan University, Wuhan, China; 7 Foshan Institute of Medical Microbiology, Foshan, China

**Keywords:** Infectious diseases, Innate immunity

## Abstract

Cytokine storm induced by Severe Acute Respiratory Syndrome Coronavirus 2 (SARS-CoV-2) is a major pathological feature of Coronavirus Disease 2019 (COVID-19) and a crucial determinant in COVID-19 prognosis. Understanding the mechanism underlying the SARS-CoV-2-induced cytokine storm is critical for COVID-19 control. Here, we identify that SARS-CoV-2 ORF3a and host hypoxia-inducible factor-1α (HIF-1α) play key roles in the virus infection and pro-inflammatory responses. RNA sequencing shows that HIF-1α signaling, immune response, and metabolism pathways are dysregulated in COVID-19 patients. Clinical analyses indicate that HIF-1α production, inflammatory responses, and high mortalities occurr in elderly patients. HIF-1α and pro-inflammatory cytokines are elicited in patients and infected cells. Interestingly, SARS-CoV-2 ORF3a induces mitochondrial damage and Mito-ROS production to promote HIF-1α expression, which subsequently facilitates SARS-CoV-2 infection and cytokines production. Notably, HIF-1α also broadly promotes the infection of other viruses. Collectively, during SARS-CoV-2 infection, ORF3a induces HIF-1α, which in turn aggravates viral infection and inflammatory responses. Therefore, HIF-1α plays an important role in promoting SARS-CoV-2 infection and inducing pro-inflammatory responses to COVID-19.

## Introduction

The infection of severe acute respiratory syndrome coronavirus 2 (SARS-CoV-2) causes Coronavirus Disease 2019 (COVID-19) and has prevalence by affecting billions of people around the world.^1,2^ Cytokine storm was reported as the main feature in the severity of COVID-19.^3,4^ Age, sex, and preexisting comorbidities, including cancer, diabetes, and cardiovascular diseases, are the main risk factors for COVID-19 patients.^5,6^ The metabolic pathways, especially the glucose metabolism, play important roles in the outcomes of COVID-19 patients. ^7^ The common approaches for COVID-19 treatments include anti-infections, anti-inflammation, and anti-cytokine storm therapies.^8^ However, there are no specific and effective drugs for the treatment of viral infection. Therefore, it is urgently needed to understand the molecular mechanisms underlying SARS-CoV-2 infection and pathogenesis as well as host immune and inflammatory responses.

To balance anti-inflammatory and pro-inflammatory responses, the host innate immunity has to ensure proper homeostasis of its defense systems.^9^ The host immune system has developed mechanisms of immune tolerance to avoid the destruction of tissues, however, it also has significant impacts on the initiation and progression of immune diseases.^10^ Strong cytokine storm is elicited in COVID-19 patients, suggesting that cytokine storm is a major risk in SARS-CoV-2 infection.^3,4^ The hypoxia-inducible factor 1α (HIF-1α) acts as a key regulator in physiological functions including metabolism, cell proliferation, and angiogenesis.^11–13^ HIF-1α is also an important activator in glycolysis and inflammatory response, which implies the effects of HIF-1α on the pathogenesis of COVID-19.^14,15^ Therefore, it is of high concern to understand SARS-CoV-2 pathogenesis and to seek potential antiviral drugs.

In this study, based on RNA sequencing and clinical specimens’ analyses, we initially showed that the expressions of HIF-1α and pro-inflammatory cytokines were induced in COVID-19 patients, and elderly patients display excessive inflammatory responses that were associated with high mortality. Notably, HIF-1α and inflammatory cytokines were induced in SARS-CoV-2-infected human cell lines. Interestingly, SARS-CoV-2 ORF3a protein promoted HIF-1α production through inducing mitochondrial damage and mitochondria-derived reactive oxygen species (Mito-ROS) production. HIF-1α subsequently enhanced the virus infection and pro-inflammatory responses. Moreover, HIF-1α could promote broadly the infections of other viruses. Therefore, we proposed that upon SARS-CoV-2 infection, ORF3a induces Mito-ROS production to activate HIF-1α, which in turn enhances the viral infection and aggravates inflammatory responses.

## Results

### Immune and metabolism pathways are dysregulated in patients with COVID-19

The mechanism by which SARS-CoV-2 interrupts the host innate immune responses is of high concern. Several studies have reported that different genes and pathways were dysregulated in patients’ specimens, including blood and Broncho alveolar lavage fluid.^9,16^ Transcriptome analysis is a useful approach to identify the dysregulated genes and pathways with different biological functions. To explore altered genes and pathways mediated by SARS-CoV-2 infection, here we performed whole-transcriptome RNA sequencing (RNA-Seq) analyses. Peripheral blood monocytes (PBMCs) were isolated and prepared from blood specimens collected from 11 COVID-19 patients (Male = 6, Female = 5; Young group with an age of 35–50 years, Elderly with an age of 65–90 years) and 9 healthy individuals (Male = 5, Female = 4; Young group with an age of 35–50 years, Elderly with an age of 65–90 years) admitted to the Tongji Hospital, Huazhong University of Science and Technology, Wuhan, China (Supplementary Table 1, 2).

**Fig. 1:**
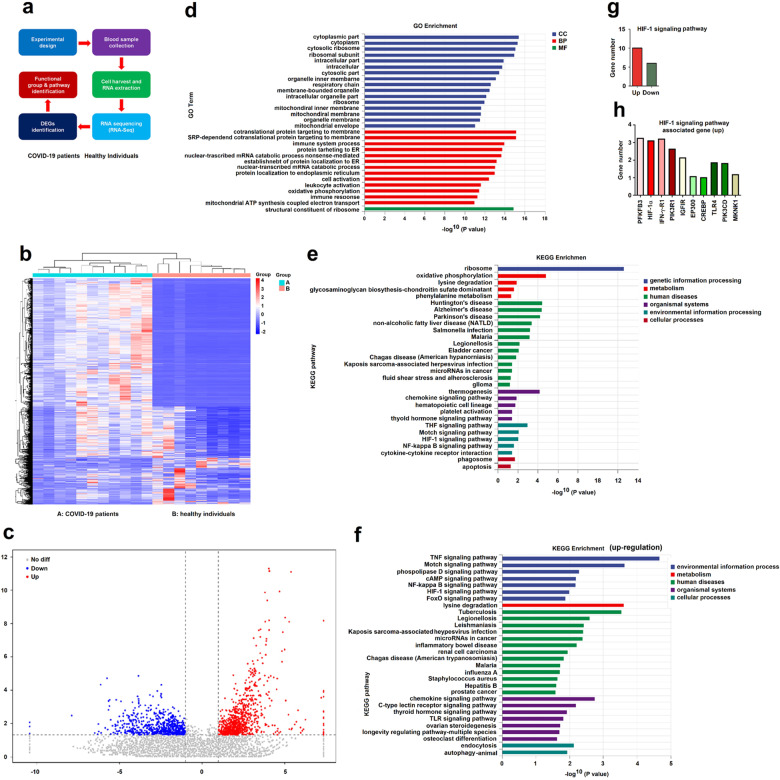
Immune and metabolism pathways are dysregulated in COVID-19 patients. **a** A basic flow chart starting from experimental design to functional groups and pathways identification. **b**, **c** Hierarchical clustering heatmap (**b**) and Volcano map (**c**) of a distinguishable mRNA expression profiling in PBMCs isolated from blood specimens of COVID-19 patients and healthy individuals. **d** Histogram description of gene ontology enrichment of DEGs, assigned to three classes as above. **e** List of the pathway enrichment (top 30) involved; enrichment scores are based as (**c**). **f** List of the upregulated pathway enrichment (top 30) involved. **g** Number of dysregulation genes of HIF-1 signaling between COVID-19 patients and healthy individuals. **h** List and fold change of up-regulation genes in HIF-1 signaling between COVID-19 patients and healthy individuals

Total mRNAs were prepared from the PBMCs isolated from whole blood samples and then subjected to RNA-Seq analyses (Fig. 1a). Among the 18,483 genes identified from the RNA-seq data, 659 genes were down-regulated and 856 genes were upregulated (Supplementary Fig. 1a). The differentially expressed genes among patients and healthy individuals were represented by Scaled Heatmap and Volcano map (Fig. 1b, c).

As the genome dysregulation was a molecular phenotype of SARS-CoV-2-infected PBMCs, we presented the top altered genes’ basic functions and dispensed them into three classes: (1) Biological process (BP), (2) Cellular component (CC), and (3) Molecular function (MF); along with 30 functional terms and significance scores. The biological processes of enriched GO terms included the membrane function, immune response, endoplasmic reticulum localization, and metabolism pathway (Fig. 1d and Supplementary Fig. 1b).

The altered pathways associated with dysregulated genes between patients and healthy individual PBMCs according to the RNA-seq data were also displayed (Fig. 1e and Supplementary Fig. 1c). We noticed that the signaling pathways of metabolism, immune, and cytokine were regulated upon SARS-CoV-2 infection (Fig. 1f). Notably, TNF signaling, NF-κB signaling, HIF-1 signaling, metabolism, and immune pathways were upregulated upon the virus infection (Fig. 1f and Supplementary Fig. 1d). Collectively, our data suggest that SARS-CoV-2 infection regulates HIF-1 signaling, immune responses, and metabolic processes metabolism pathways.

Previous studies reported that age is a risk factor among COVID-19 patients.^5,6^ Here, we evaluated the link between age and inflammatory response. The differentially expressed genes in the RNA-Seq data were presented in scaled heatmaps (Supplementary Fig. 2a, Supplementary Table 2). The enriched GO terms showed that the immune pathway and cytokines were enriched in elderly individuals with an age of 65–90 years (Supplementary Fig. 2c), but not in young individuals with an age of 35–50 years (Supplementary Fig. 2b). Notably, HIF-1 signaling, IL-17 signaling, and immune pathways were enriched in the elderly group (Supplementary Fig. 2e), but not in the young group (Supplementary Fig. 2d). Moreover, HIF-1 signaling and immune pathways were upregulated in the elderly group (Supplementary Fig. 2f). As HIF-1 signaling is a strong inducer in glycolysis and inflammatory response,^12^ our data suggests that HIF-1 signaling is one of the key regulators in elderly patients associated with inflammatory responses. Notably, 10 genes in the HIF-1α pathway, including the hypoxia-inducible factor-1α (HIF-1α), were upregulated in the PBMCs of COVD-19 patients Notably, 10 genes in the HIF-1 signaling pathway, including the hypoxia-inducible factor-1α (HIF-1α) and 6-phosphofructo-2-kinase/fructose-2, 6-bisphosphatase 3 (PFKFB3), two glycolysis association genes, and other genes associated with cell proliferation, immune response, and metabolism were upregulated in the PBMCs of COVD-19 patients (Fig. 1g, h). Collectively, our data suggest that SARS-CoV-2 infection is highly associated with the pathways of HIF-1α, immune, and metabolism in COVID-19 patients’ blood samples.

### Elderly COVID-19 patients display excessive inflammatory responses and high mortality

**Fig. 2:**
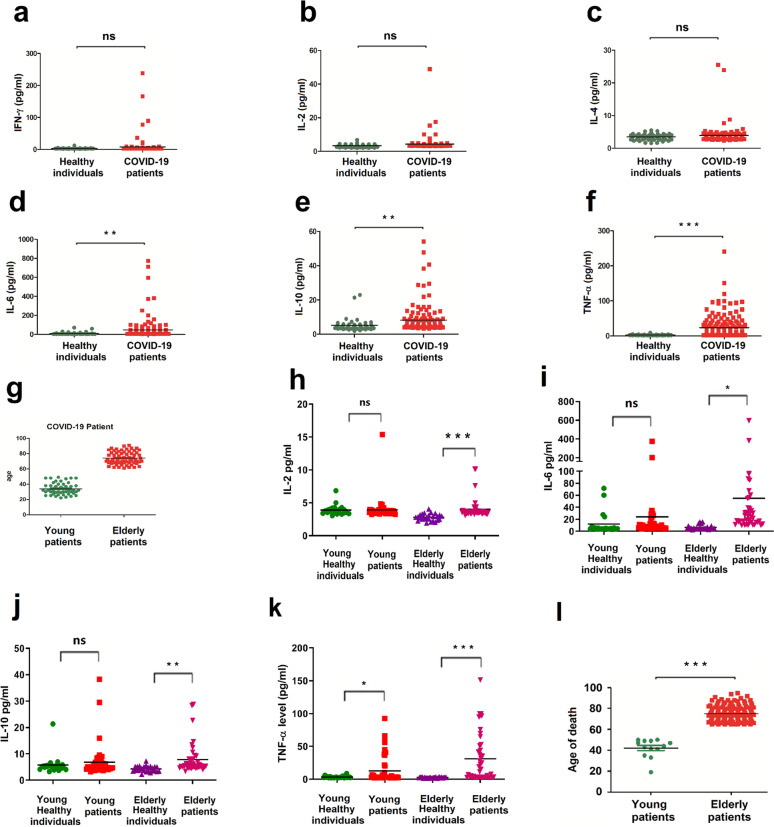
Elderly patients display excessive inflammatory responses and high mortality. **a**–**f** Blood samples from healthy individuals (*n* = 65) and COVID-19 patients (*n* = 143) admitted to the Renmin Hospital, Wuhan, China, were collected. IFN-γ, IL-2, IL-4, Il-6, IL-10, and TNF-α proteins expressed in the sera were determined by flow cytometry. **g** Among 143 patients, 49 were young with an age of 20–50 years (average age = 33.4 years), and 64 were old with an age of 61–90 years (average age = 74.1 years) (61–90 years). **h**–**k** Blood samples were collected from healthy individuals (*n* = 65) and COVID-19 patients (*n* = 143) admitted to the Renmin Hospital, Wuhan University, Wuhan, China (Supplementary Table 3), young healthy individuals (*n* = 22), young patients (*n* = 49) (20–50 years), 25 elderly healthy individuals and 44 elderly patients (61–79 years) were sift out. IL-2, IL-6, IL-10, and TNF-α proteins expressed in the sera were determined by flow cytometry. **l** Among the 222 died COVID-19 patients, 150 patients with age over 65 years (elderly patients), and 13 patients with age under 50 years (young patients)

The roles of SARS-CoV-2 infection in proteins’ productions involved in the immune and inflammatory responses were further evaluated in patients. Blood samples were collected from healthy individuals (*n* = 65) and COVID-19 patients (*n* = 143) admitted to the Renmin Hospital, Wuhan University, Wuhan, China (Supplementary Table 3). The results showed that IFN-γ, IL-2, and IL-4 protein levels were no significant differences in PBMCs of the patients’ group as compared to the healthy individuals’ group (Fig. 2a–c). However, the levels of IL-6, IL-10, and TNF-α proteins were remarkably induced in PBMCs of the patients’ group as compared to the healthy group (Fig. 2d–f), suggesting that SARS-CoV-2 elicits inflammatory responses in patients’ PBMCs.

Because SARS-CoV-2 susceptibility and COVID-19 clinical outcomes are different in young and elderly patients, elderly patients with COVID-19 are more likely to progress to severe diseases.^17–19^ We next evaluated the effects of SARS-CoV-2 on immune and inflammatory responses between young and elderly COVID-19 patients. Notably, among 143 patients, 49 were young with an age of 20–50 years (average age = 33.4 years) and 64 were old with an age of 61–90 years (average age = 74.1 years) (Fig. 2g), suggesting that elderly individuals are more susceptible to the infection of SARS-CoV-2. Among the healthy individuals, 22 were young with an age of 20–50 years (average age = 33.7 years) and 25 were old with an age of 61–79 years (average age = 66.4 years); and among the patients, 49 were young with an age of 20–50 years (average age = 33.4 years), and 44 were old with an age of 61–79 years (average age = 69.3 years). Furthermore, we showed that no significant differences in secreted IFN-γ and IL-4 proteins between the elderly patient group and the young patient group were detected (Supplementary Fig. 3a, b). The levels of secreted IL-2, IL-6, and IL-10 proteins were no different in the young patients related to the young healthy individuals, but they were significantly notably higher in the elderly patients related to the elderly healthy individuals (Fig. 2h–j). The level of TNF-α protein was slightly upregulated in the young patients related to the young healthy individuals, but notably higher in the elderly patients related to the elderly healthy individuals (Fig. 2k). These results suggest that SARS-CoV-2 infection provokes excessive pro-inflammatory cytokines in elderly patients.

Moreover, the correlation of age and death in COVID-19 patients was evaluated. We noted that the numbers of death cases were much higher in patients of age over 65 years (*n* = 150) as compared to patients of age less than 50 years (*n* = 13) (Fig. 2l and Supplementary Table 4). Remarkably, stochastic analyses showed that among 222 died COVID-19 patients: (1) the ratio of male (*n* = 151) to female (*n* = 71) = 2.1:1; (2) the average age of died patients = 68.8 years old; (3) 96 died patients had chronic diseases such as cancer, diabetes, kidney diseases, liver diseases and cardiovascular diseases, and (4) the average death time (the time from onset to death) was 20 days (Supplementary Table 5). Collectively, these data indicated that excessive inflammatory responses are elicited in elderly patients that may cause the severity of the diseases and the death of the patients.

### HIF-1α and pro-inflammatory cytokines are induced upon SARS-CoV-2 infection

**Fig. 3:**
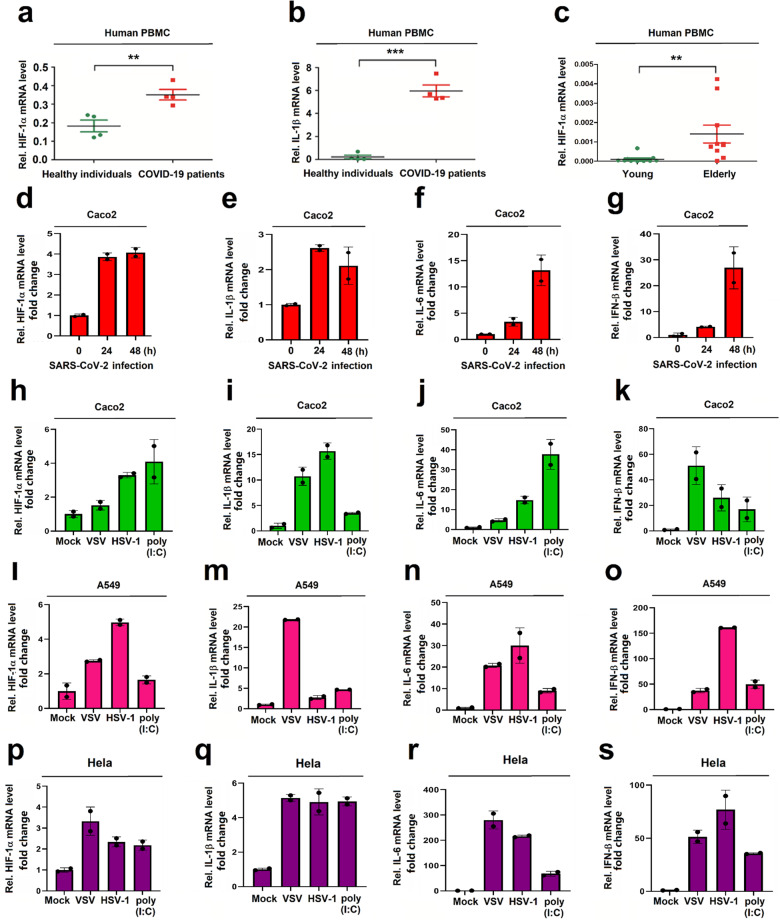
HIF-1α and inflammatory cytokines are induced by SARS-CoV-2 infection. **a**, **b** Blood samples from COVID-19 patients (*n* = 4) and healthy individuals (*n* = 4) admitted to the Tongji Hospital, Wuhan, China, were collected. The RNAs were extracted from the sera. HIF-1α mRNA (**a**) and IL-1β mRNA (**b**) was determined by RT-PCR. **c** Blood samples from young healthy individuals (*n* = 9) and elderly healthy individuals (*n* = 9) admitted to the Tongji Hospital, Wuhan, China, were collected. The RNAs were extracted from the sera. HIF-1α mRNA was determined by RT-PCR. **d**–**g** Caco2 cells were infected with SARS-CoV-2 for 24 and 48 h. **h**–**k** Caco2 cells were infected with VSV or HSV-1 or treated with poly(I: C). **l**–**o** A549 cells infected with VSV or HSV-1 or treated with poly(I: C). **p**–**s** HeLa cells were infected with VSV or HSV-1 or treated with poly(I: C). **d**–**s** The levels of HIF-1α, IL-1β, IL-6, and IFN-β mRNAs were determined by qRT-PCR

The molecular mechanism underlying SARS-CoV-2-induced immune-inflammatory responses were evaluated. As our RNA-Seq data showed that the regulation of HIF by oxygen pathway and the HIF-1α signaling pathway were upregulated in the blood samples of COVD-19 patients, we speculated that HIF-1α plays an important role in regulating immune and inflammatory responses upon the viral infection. We showed that HIF-1α mRNA level was much higher in PBMCs of patients related to healthy individuals (Fig. 3a). We also noted that similarly to HIF-1α, the level of IL-1β mRNA was much higher in PBMCs of COVID-19 patients related to healthy individuals (Fig. 3b). Considering higher levels of inflammatory cytokines in the elderly patients group as compared to the young ones, the correlation of age with HIF-1α expression was evaluated in healthy individuals. Notably, HIF-1α mRNA level was remarkably higher in PBMCs of elderly healthy individuals as compared to the young healthy individuals (Fig. 3c). Collectively, the data suggested that HIF-1α expression is induced upon SARS-CoV-2 infection, correlated well to IL-1β expression, and is highly stimulated in elderly individuals.

Next, the roles of SARS-CoV-2 in the expression of HIF-1α and inflammatory cytokines were evaluated in cultured cells. A permissive SARS-CoV-2 infection model, human colon cancer cell (Caco2), was established based on a previous report.^16^ SARS-CoV-2 nucleoprotein (N) mRNA and ORF1a mRNA were detected in infected Caco2 cells at 24 and 48 h post-infection (h.p.i) (Supplementary Fig. 4a, b). Similarly, SARS-CoV-2 N protein was produced in infected Caco2 cells at 24 h.p.i (Supplementary Fig. 4c). Cytopathic effect (CPE) was also observed in infected cells but not in uninfected cells (Supplementary Fig. 4d). The results confirmed that SARS-CoV-2 favorably infects and robustly replicates in Caco2 cells. Notably, HIF-1α, IL-1β, IL-6, and IFN-β mRNAs and HIF-1α protein were induced in infected cells (Fig. 3d–g and Supplementary Fig. 4e), suggesting that HIF-1α and immune and inflammatory cytokines are induced upon the virus infection, and HIF-1α expression is linked to immune-inflammatory cytokine expression in infected cultured cells.

Moreover, the correlation of HIF-1α with immune-inflammatory cytokines was further determined using different stimuli. Caco2 cells were infected with vesicular stomatitis virus (VSV) (an RNA virus) or herpes simplex virus-1 (HSV-1) (a DNA virus) or treated with poly(I: C) (a dsRNA analogue). HIF-1α, IL-1β, IL-6, and IFN-β mRNAs and HIF-1α protein were induced in Caco2 cells by VSV, HSV-1, or poly(I: C) (Fig. 3h–k and Supplementary Fig. 4f), indicating that HIF-1α production is associated with cytokine expression in VSV- or HSV-1-infected or poly(I: C)-stimulated Caco2 cells. In human alveolar basal epithelial cells (A549), VSV infection, HSV-1 infection, or poly(I: C) treatment activated HIF-1α, IL-1β, IL-6, and IFN-β mRNAs (Fig. 3l–o). Similarly, in human cervical carcinoma cells (Hela), HIF-1α, IL-1β, IL-6, and IFN-β mRNAs were promoted by VSV infection, HSV-1 infection and poly(I: C) treatment (Fig. 3p–s) and HIF-1α protein was induced by VSV infection, HSV-1 infection or poly(I: C) treatment (Supplementary Fig. 4g). Moreover, in human acute monocytic leukemia (THP-1) cells, VSV infection, HSV-1 infection, or LPS treatment activated HIF-1α expression (Supplementary Fig. 4h). Taken together, we demonstrated that HIF-1α is induced in PBMCs of patients, and activated upon SARS-CoV-2, VSV, or HSV-1 infection or by poly(I: C) treatment in human cell lines. We also revealed that HIF-1α expression is highly and positively correlated to immune-inflammatory cytokines production upon SARS-CoV-2, VSV, or SV-1 infection or by poly(I: C) treatment.

### ORF3a promotes HIF-1α production and inflammatory responses via Mito-ROS signaling

**Fig. 4:**
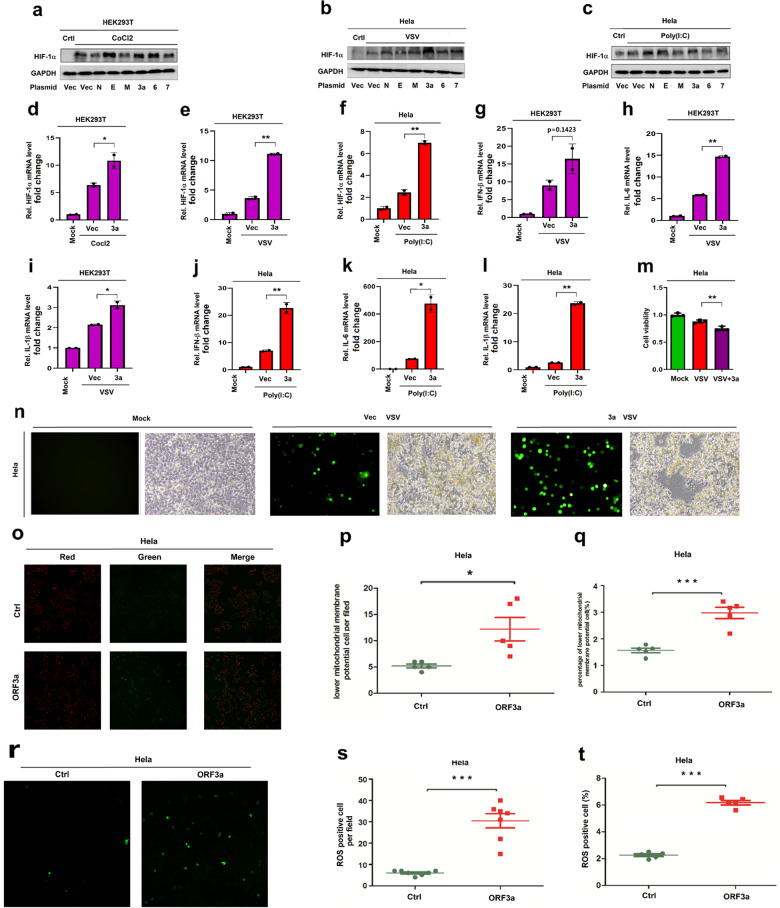
SARS-CoV-2 ORF3a promotes HIF-1α production and inflammatory responses. **a** Human HEK293T cells were transfected with indicated plasmids for 24 h, and then treated with CoCl_2_ for 6 h. HIF-1α and GAPDH proteins were assessed by WB. **b**, **c** Hela cells were transfected with indicated plasmids for 24 h, and then infected with VSV for 24 h or treated with poly(I: C) for 12 h. HIF-1α and GAPDH proteins were determined by WB. **d**, **e** HEK293T cells were transfected with indicated plasmids for 24 h, and then treated with CoCl_2_ for 6 h or infected with VSV for 12 h. HIF-1α mRNA was determined by RT-PCR. **f** Hela cells were transfected with indicated plasmids for 24 h, and then treated with poly(I: C) for 12 h. HIF-1α mRNA was determined by RT-PCR. **g**–**i** HEK293T cells transfected with indicated plasmids for 24 h, and then infected with VSV for 24 h, IL-1β, IL-6, and IFN-β mRNAs were analyzed by RT-PCR. **j**–**l** Hela cells were transfected with indicated plasmids and then treated with poly(I: C) for 12 h, IL-1β, IL-6, and IFN-β mRNAs were analyzed by RT-PCR. **m**, **n** Hela cells were transfected with indicated plasmids for 24 h and then infected with VSV 24 h. Cell viability was determined (**m**) and cell images showing the virus infection and cell cytotoxicity (**n**). **o**–**q** Cells were strained with JC-1 Kit (Beyotime, C2006) according to the provided instructions. Mitochondrial membrane potentials were analyzed by confocal microscopy, the red parts represented normal mitochondrial membrane potentials, green parts represented lower mitochondrial membrane potentials (**o**). Five filed were calculation the lower mitochondrial membrane positive cell number (**p**). Mitochondrial membrane potentials were analysed by Flow cytometry (**q**). **r**–**t** Cells were strained with ROS Kit (Beyotime, S0033S) according to the provided instructions. ROS level was analysed by confocal microscopy, green part represents ROS positive cell (**r**). Six filed were calculation the ROS positive cell number (s). ROS positive cell was analysed by Flow cytometry (**t**)

The mechanism underlying HIF-1α expression and inflammatory responses induced by SARS-CoV-2 was further explored. According to the sequences of the SARS-CoV-2 genome,^20^ we designed and synthesized the virus genes encoding for three structural proteins: membrane protein (M), an envelope protein (E), and nucleocapsid protein (N), as well as three accessory proteins: ORF3a, ORF6, and ORF7. The productions of these proteins were subsequently detected and confirmed (Supplementary Fig. 5a). The roles of these genes participated in the regulation of HIF-1α expression and proinflammatory responses were determined. In HEK293T cells transfected with plasmids carrying the virus and treated with CoCl_2_ (a HIF-1α inducer),^21–23^ HIF-1α protein was induced by CoCl_2_ treatment and further enhanced by SARS-CoV-2 E, ORF3a, and ORF6 proteins (Fig. 4a). Similarly, in Hela cells transfected with plasmids carrying the viral genes and infected with VSV or treated with poly(I: C), HIF-1α protein was induced upon VSV infection and further enhanced by SARS-CoV-2 ORF3a protein (Fig. 4b), while HIF-1α protein was stimulated by poly(I: C) treatment and further facilitated by SARS-CoV-2 ORF3a, N, and E proteins (Fig. 4c). The role of SARS-CoV-2 ORF3a in promoting HIF-1α expression was further assessed. We showed that ORF3a enhanced HIF-1α mRNA expression in CoCl_2_-treated HEK293T cells (Fig. 4d), VSV-infected HEK293T cells (Fig. 4e), and poly(I: C)-treated Hela cells (Fig. 4f). Collectively, these results illustrate that HIF-1α protein production is facilitated by SARS-CoV-2 ORF3a, implying that ORF3a plays role in regulating proinflammatory responses.

Next, the role of SARS-CoV-2 ORF3a in regulating proinflammatory cytokine production was evaluated. The results showed that IFN-β, IL-6, and IL-1β mRNAs were induced upon VSV infection and further promoted by ORF3a in HEK293T cells (Fig. 4g–i). Similarly, IFN-β, IL-6, and IL-1β mRNAs were induced by poly(I: C) and further enhanced by ORF3a in Hela cells (Fig. 4j–l). Notably, in primary human umbilical vein endothelial cells (HUVECs), HIF-1α protein was stimulated by poly(I: C) and further facilitated by ORF3a (Supplementary Fig. 5b), and IFN-β mRNA as well as IL-6 mRNAs were induced by poly(I: C) and further promoted by ORF3a (Supplementary Fig. 5c), indicating that ORF3a promotes HIF-1α production and inflammatory cytokine expression in primary human cells. Collectively, our results demonstrate that ORF3a facilitates proinflammatory cytokine production through activating HIF-1α.

Moreover, the effect of SARS-CoV-2 ORF3a on the regulation of VSV replication and infection through inducing HIF-1α production and proinflammatory cytokine production was evaluated. Remarkably, ORF3a reduced cell viability in VSV-infected Hela cells (Fig. 4m), promoted VSV replication in Hela cells (Fig. 4n, left), and facilitated cytotoxicity in VSV-infected Hela cells (Fig. 4n, right), suggesting that ORF3a facilitates VSV replication and infection. We also noticed that in HUVECs, HIF-1α protein was induced upon VSV infection and further promoted by ORF3a (Supplementary Fig. 5d) and that VSV replication was enhanced by ORF3a (Supplementary Fig. 5e), demonstrating that ORF3a promotes HIF-1α production in human primary cells. Moreover, ORF3a promoted VSV replication, but this promotion was repressed by BAY87-2243 (a HIF-1α inhibitor) in Hela cells (Supplementary Fig. 6a–c). These data indicate that HIF-1α has a broad role in promoting viral replication. Taken together, the results suggest that ORF3a promotes HIF-1α production, inflammatory responses, and virus infection.

The mechanism of SARS-CoV-2 ORF3a promoting HIF-1α expression and facilitating inflammatory responses was further explored. The results showed that ORF3a was located in the cytoplasm in mock-infected, HSV-1-infected, and VSV-infected Hela cells (Supplementary Fig. 6d), indicating that ORF3a location is not affected by HSV-1 or VSV. Previous studies reported that Mitochondria-derived reactive oxygen species (Mito-ROS) is a strong inducer for HIF-1α and that SARS-CoV-2 induces ROS and HIF-1α production.^15^ Here, we explored whether ORF3a regulates HIF-1α through Mito-ROS. The results showed that mitochondrial membrane potentials were reduced by ORF3a (Fig. 4o–q), while ROS generations were promoted by ORF3a (Fig. 4r–t), indicating that ORF3a induces mitochondrial damages. Altogether, our results suggest that ORF3a promotes HIF-1α production, immune-inflammatory responses and virus infection through inducing the Mito-ROS signaling.

### HIF-1α promotes SARS-CoV-2 infection and inflammatory responses

Given the essential roles of HIF-1α in biological processes,^24–26^ we speculated that HIF-1α may also play important role in regulating SARS-CoV-2 replication and immune-inflammatory responses. Initially, we determined the effect of HIF-1α on regulating the virus replication and the production of inflammatory cytokines. In Hela cells, HIF-1α protein was induced by CoCl_2_ or poly(I: C), whereas such induction was repressed by BAY87-2243 (Supplementary Fig. 7a, b) in a considerable dose without cytotoxicity (Supplementary Fig. 7c). Similarly, in Caco2 cells, poly(I: C)-induced HIF-1α protein was repressed by BAY87-2243 in a dose-dependent fashion (Supplementary Fig. 7d), with no effect on Caco2 cells’ viability (Supplementary Fig. 7e). In HUVECs, HIF-1α protein was stimulated by poly(I: C), but poly(I: C)-induced HIF-1α protein was repressed by BAY87-2243 (Supplementary Fig. 7f).

**Fig. 5:**
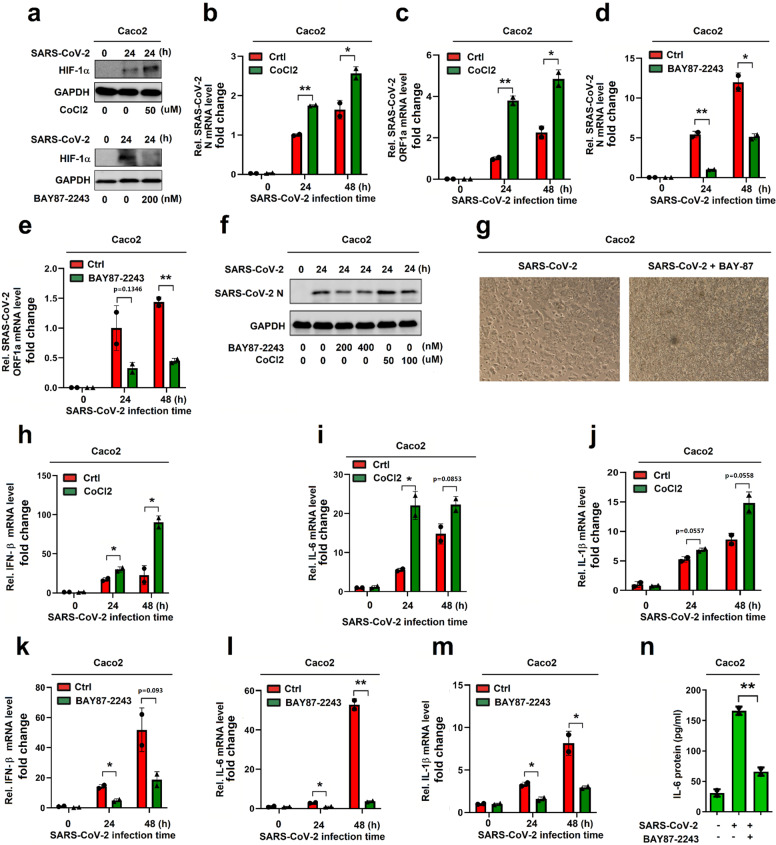
HIF-1α promotes SARS-CoV-2 infection and inflammatory responses. **a** Caco2 cells were treated with CoCl_2_ or BAY87-2243 and then infected with SARS-CoV-2 for 24 h. HIF-1α and GAPDH proteins were assessed by WB. **b**, **c** Caco2 cells were treated with CoCl_2_ and infected with SARS-CoV-2 for 24 and 48 h. The levels of SARS-CoV-2 N mRNA and SARS-CoV-2 ORF1a mRNA were analyzed by RT-PCR. **d**, **e** Caco2 cells were treated with BAY87-2243, following infection with SARS-CoV-2 for indicated times. SARS-CoV-2 N and ORF1a mRNAs were determined by RT-PCR. **f** BAY87-2243- or CoCl_2_-treated Caco2 cells were infected with SARS-CoV-2. SARS-CoV-2 N protein and GAPDH protein were analyzed by WB. **g** BAY87-2243-treated Caco2 cells were infected with SARS-CoV-2 for 48 h. Cell images showing the cell cytotoxicity. **h**–**j** CoCl_2_-treated Caco2 cells were infected with SARS-CoV-2 for indicated times. The levels of IL-1β, IL-6, and IFN-β mRNAs were detected by RT-PCR. **k**–**n** Caco2 cells were treated with BAY87-2243 and then infected with SARS-CoV-2 for 24 and 48 h. The levels of IL-1β, IL-6, and IFN-β mRNAs were analyzed by RT-PCR (**k**–**m**), and the level of IL-6 protein was determined by ELISA (**n**)

Next, the effect of HIF-1α on regulating the viral replication was then assessed. Notably, in Caco2 cells infected with SARS-CoV-2, HIF-1α protein was promoted by CoCl_2_ (Fig. 5a, top), but attenuated by BAY87-2243 (Fig. 5a, bottom). Correspondingly, SARS-CoV-2 N and ORF1a mRNAs were enhanced by CoCl_2_ (Fig. 5b, c) and repressed by BAY87-2243 (Fig. 5d, e). We also observed a similar result of SARS-CoV-2 N protein production (Fig. 5f) and noted that cell cytotoxicity was significantly reduced by BAY87-2243 in Caco2 cells infected with SARS-CoV-2 (Fig. 5g). The results confirmed that HIF-1α plays a positive role in regulating SARS-CoV-2 replication.

Moreover, the effect of HIF-1α on regulating inflammatory responses was explored. IL-1β protein was induced by LPS, but LPS-induced IL-1β was repressed by BAY87-2243 (Supplementary Fig. 7g). IL-6 mRNA was induced by VSV, poly(I: C), poly(dA:dT), and LPS, while this induction was repressed by BAY87-2243 (Supplementary Fig. 7h). The data suggest that induction of HIF-1α promotes inflammatory cytokines, whereas inhibition of HIF-1α represses inflammatory cytokines. We also noticed that IFN-β, IL-6, and IL-1β mRNAs were induced upon SARS-CoV-2 infection and further enhanced by CoCl_2_ (Fig. 5h–j), but repressed by BAY87-2243 (Fig. 5k–m); and IL-6 protein was induced upon SARS-CoV-2 infection but repressed by BAY87-2243 (Fig. 5n). Collectively, these data demonstrated that induction of HIF-1α promotes the expression of inflammatory cytokines, whereas inhibition of HIF-1α represses the production of inflammatory cytokines. Taken together, these results suggest that HIF-1α promotes SARS-CoV-2 replication and immune-inflammatory responses in infected cells.

### HIF-1α plays extensive roles in facilitating viruses’ infections

**Fig. 6:**
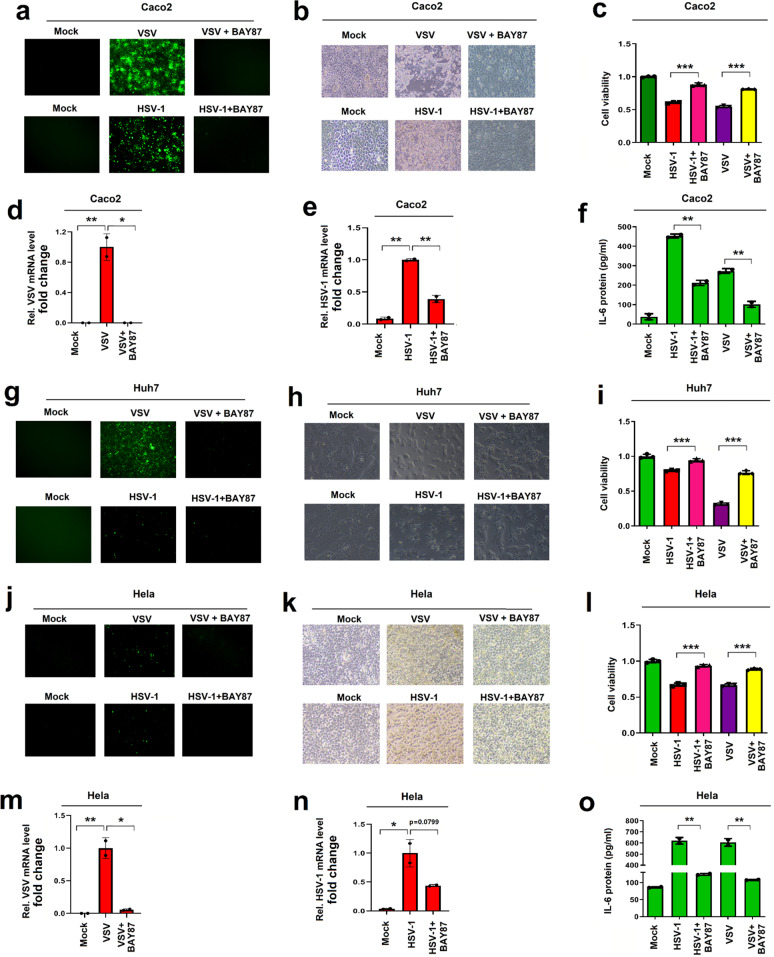
HIF-1α plays an extensive role in facilitating the infections of viruses. **a**–**c** Caco2 cells were treated with BAY87-2243 and infected with VSV (top) or HSV-1 (bottom) for 24 h. Images showing virus infection (**a**), cell cytotoxicity (**b**), and cells viability (**c**) by the cell counting kit-8. **d**–**f** BAY87-2243-treated Caco2 cells were infected with VSV or HSV-1 for 24 h. The mRNA levels of VSV and HSV-1 were analyzed by RT-PCR (**d**, **e**). The protein level of IL-6 was analyzed by ELISA (**f**). **g**–**i** Huh7 cells were treated with BAY87-2243 and then infected with VSV or HSV-1 for 24 h. Cell images showing the virus infection (**g**), cell cytotoxicity (**h**), and cell viability (**i**) were assessed by the cell counting kit-8. **j**–**o** Hela cells were treated with BAY87-2243 and then infected with VSV or HSV-1 for 24 h. Cell images showing the virus infection (**j**), cell cytotoxicity (**k**), and cells viability (**l**) were assessed by the cell counting kit-8. The mRNA levels of VSV and HSV-1 were analyzed by RT-PCR (**m**, **n**). The protein level of IL-6 was analyzed by the ELISA (**o**)

As HIF-1α promotes SARS-CoV-2 replication and is induced upon infections of various viruses including SARS-CoV-2, VSV, and HSV-1, and thus, we determined the effect of HIF-1α on the infections of other viruses. Notably, VSV and HSV-1 replicated robustly in Caco2 cells, but the viral replications were repressed by BAY87-2243 (Fig. 6a). Accordingly, VSV- or HSV-1-mediated inductions of cytotoxicity were notably reduced by BAY87-2243 (Fig. 6b), and VSV- or HSV-1-mediated reductions of cell viability were suppressed by BAY87-2243 (Fig. 6c). Remarkably, in infected Caco2 cells, the RNA levels of VSV (Fig. 6d) and HSV-1 (Fig. 6e) were inhibited by BAY87-2243. We also noted that VSV- and HSV-1-induced IL-6 protein was attenuated by BAY87-2243 in Caco2 cells (Fig. 6f). Similarly, in infected Huh7 cells, VSV or HSV-1 replications were repressed by BAY87-2243 (Fig. 6g), VSV or HSV-1-induced cytotoxicity was significantly attenuated by BAY87-2243 (Fig. 6h), and VSV- or HSV-1-mediated reduction of cell viability was recovered by BAY87-22437 (Fig. 6i). Taken together, the data demonstrated that inhibition of HIF-1α results to the repression of VSV and HSV-1 replication.

Consistently, in infected Hela cells, BAY87-2243 repressed the infections of VSV and HSV-1 (Fig. 6j), reduced viral-induced cell cytotoxicity (Fig. 6k), and recovered viral-mediated cell viability (Fig. 6l). Remarkably, in infected Hela cells, BAY87-2243 could inhibit VSV mRNA production (Fig. 6m), suppress HSV-1 mRNA expression (Fig. 6n), and repress VSV- and HSV-1-induced IL-6 protein production (Fig. 6o). Therefore, these results demonstrate that inhibition of HIF-1α leads to the repression of the infections of VSV and HSV-1 and the production of inflammatory cytokines.

## Discussion

The emerging SARS-CoV-2 leading to the COVID-19 pandemic has become a great threat to public health globally.^27–29^ Host-based treatments such as anti-inflammation, anti-infection, or anti-cytokine storm are the common strategies used to treat or mitigate the spread of SARS-CoV-2.^8^ Blocking systemic inflammation and inhibiting SARS-CoV-2 infection becomes two major promising strategies for COVID-19 treatment.^30,31^ Until now, there are no specific target drugs available for COVID-19 treatment, therefore, seeking new potential targets for antiviral infection is of high concern. The SARS-CoV-2 infection causes dysfunctional immune responses, which makes patients suffer from severe systemic inflammation.^32^ Many studies have reported that SARS-CoV-2 infection dysregulates host immune responses to induce excessive inflammatory cytokines, known as the cytokine storm, thereby contributing to the severity and poor prognosis of the illness.^33–35^ Multiple metabolic disorders play a positive role in SARS-CoV-2 pathogenesis in COVID-19 progression.^36,37^ The molecular mechanisms underlying SARS-CoV-2 pathogenesis need further to be determined. Therefore, it is of high significance to identify the key factors responsible for modulating immune and inflammatory responses upon SARS-CoV-2 infection, which may help clinical treatment.

Transcriptome analyses are useful approaches to identify the altered genes and related pathways with many biological functions.^38^ In the current study, we performed whole-transcriptome RNA sequencing analyses with PBMCs isolated from COVID-19 patients and healthy individuals. The data showed that SARS-CoV-2 infection significantly affects the immune response, metabolic pathway, cell death, cell cycle, viral responses, and apoptotic process. Notably, both the regulation of HIF by oxygen pathway and the HIF-1α signaling pathway were also dysregulated in the PBMCs of COVD-19 patients.

In addition, the analyses of clinical specimens indicated that the expressions of HIF-1α and inflammatory cytokines were remarkably elicited in COVID-19 patients in comparison with healthy individuals, significantly provoked in elderly patients as compared to young patients, and was also elevated in elderly healthy individuals as compared to young healthy individuals. Previous studies reported that the mortality of COVID-19 increases exponentially with the increase of age.^17^ In accordance with this, we noted that death cases were much more in patients of age over 61 years as compared to patients of age less than 60 years. Collectively, these data indicate that HIF-1α and excessive inflammatory responses elicited in elderly patients may lead to disease severity and patient death.

The effects of SARS-CoV-2 infection on HIF-1α expression and inflammatory cytokines’ production were evaluated in cultured cells. We noted that HIF-1α, IL-1β, IL-6, and IFN-β were significantly induced in Caco2 cells infected with SARS-CoV-2, consistent with a recent report showing induction of HIF-1α in SARS-CoV-2-infected cells^39^. Collectively, our results demonstrated that HIF-1α was induced in COVID-19 patients, elicited significantly in elderly patients, and activated upon the infections of SARS-CoV-2, VSV, or HSV-1 or the treatment of poly(I: C) in cultured cells. Notably, the expression of HIF-1α was positively correlated with the production of immune and inflammatory cytokines upon the infection of SARS-CoV-2, VSV, and HSV-1 and the treatment of poly(I: C). These data suggested that HIF-1α plays a critical role in regulating immune and inflammatory responses upon SARS-CoV-2 infection.

Based on the sequences of the SARS-CoV-2 genome,^20^ we designed and cloned 6 viral genes, and screened the candidate proteins that impact the productions of HIF-1α and proinflammatory cytokines. The results illustrated that SARS-CoV-2 ORF3a was involved in the activations of HIF-1α and proinflammatory cytokines. Previous studies showed that ORF3a causes cell death, lysosomal damage, and cell apoptosis.^40–42^ Remarkably, we noted that ORF3a could reduce cell viability, promote VSV replication, and facilitate cytotoxicity in VSV-infected cells, suggesting that ORF3a facilitates virus infection. In addition, we revealed a distinct mechanism by which ORF3a induces mitochondrial damage and Mito-ROS production to promote HIF-1α expression. Therefore, our data revealed that SARS-CoV-2 ORF3a plays important roles in regulating HIF-1α production, proinflammatory responses, and viral infection. Altogether, we presented that SARS-CoV-2 ORF3a promotes HIF-1α production to induce immune-inflammatory responses and facilitate virus infection. Further investigation is required for thoroughly uncover how SARS-CoV-2 ORF3a induces the HIF-1α pathway.

HIF-1α plays a key role in regulating metabolic pathways and inflammatory responses.^43^ Dysregulation of the HIF-1α pathway promotes multiple diseases including cancer, cardiovascular disease, and Alzheimer’s disease.^44–47^ Particularly, HIF-1α also contributes to the regulation of the aging process^47^. Given the essential roles of HIF-1α in many important biological processes, we determined the effect of HIF-1α in regulating SARS-CoV-2 replication and inflammatory responses. Notably, in SARS-CoV-2-infected Caco2 cells, virus replication, cell cytotoxicity, and cytokines production were promoted by CoCl_2_ (a HIF-1α inducer) but attenuated by BAY87-2243 (a HIF-1α inhibitor), indicating that HIF-1α promotes virus replication and immune-inflammatory responses in infected cells. As HIF-1α promotes SARS-CoV-2 replication and is induced upon the infection of several viruses, thus, we determined the effect of HIF-1α on the infection of other viruses. Remarkably, VSV and HSV-1 replication, cell cytotoxicity, and IL-6 production were repressed by BAY87-2243, whereas the reduction of cell viability was suppressed by BAY87-2243 in infected Caco2, Huh7, and Hela cells. The results demonstrated that HIF-1α plays an extensive role in facilitating virus infections and inflammatory responses.

Collectively, our research demonstrated that during SARS-CoV-2 infection, the viral ORF3a protein elevates the production of HIF-1α, which in turn promotes SARS-CoV-2 infection and inflammatory responses. Therefore, HIF-1α is a key activator for both SARS-CoV-2 infection and inflammatory response, thereby serving as a potential therapeutic target for virus-induced inflammatory diseases and COVID-19.

## Methods

### Patients sample analyses

Human blood samples of COVID-19 patients and healthy individuals analyzed in this study were collected from Tongji Hospital (Wuhan, China). This study was approved by Ethics in Human Studies. Peripheral blood mononuclear cells (PBMCs) were isolated by separating blood samples and diluting them in RPMI-1640 (Gibco; Grand Island, USA). These cells were then gently overlaid onto an equivalent volume of lymphocyte separation medium (#50494, MP Biomedicals, Santa Ana, USA) and then spun at 2000 × g for 10 min at room temperature (RT). The interface layer containing PBMCs was then collected and diluted using RPMI-1640. The total RNA from the tissues or cells were extracted with a Trizol reagent. RNA samples were used to RNA-seq, which were provided by Personal Biotechnology Co., Ltd. Shanghai, China. The data were analyzed by using the free online platform Personalbio GenesCloud (https://www.genescloud.cn). The serum was collected and analyzed the cytokine was through Flow cytometry.

### Ethics statement

This work was performed according to the principles of the Declaration of Helsinki and approved by the Institutional Review Board of the College of Life Sciences, Wuhan University, China, in accordance with its guidelines for the protection of human subjects. The Institutional Review Board of the College of Life Sciences of Wuhan University, China, approved the collection of clinical specimens for this study. Written informed consents were obtained from all participants.

### Cell lines and cultures

Human hepatocarcinoma cell line (Huh7), human non-small cell lung cancer cell line (A549), and African green monkey kidney cell line (Vero), Embryonic kidney cell line (HEK293T), and Henrietta Lacks cell line (Hela) were purchased from American Type Culture Collection (ATCC). The human umbilical vein endothelial cell line (HUVEC) and human colon adenocarcinoma cell line (Caco2) was purchased from China Type Culture Collection (CTCC), Wuhan, China. The human monocytic monocyte cell line (THP-1) was kindly provided by Professor Bing Sun of the Institute of Biochemistry and Cell Biology, Shanghai Institute for Biological Sciences, China. All cells, except THP-1 cells, were cultured in Dulbecco’s modified Eagle’s medium (DMEM) (Gibco, Grand Island, NY, USA) containing 10% Fetal Bovine Serum (FBS), 100 U/ml penicillin, and 100 μg/ml streptomycin sulfate, and then maintained and cultured at 37 °C in a 5% CO_2_ incubator. The THP-1 cells were maintained and cultured in RPMI-1640 medium supplemented with 10% FBS, 100 U/ml penicillin, and 100 μg/ml streptomycin sulfate.

### Antibodies and reagents

Antibody for Flag (F3165) (1:2000) and monoclonal antibody mouse anti-GAPDH (G9295) (1:5000) were purchased from Sigma (St Louis, MO, USA). Antibodies anti-HIF-1α (36169 s) and anti-IL-1β (12703 S) were purchased from Cell Signaling Technology. Antibodies anti-GFP (A19535) anti-SARA-COV-2 -N (ab20021) were purchased from ABclonal. Lipofectamine 2000 were purchased from Invitrogen (Carlsbad, CA, USA). CoCl_2_ were purchased from Sigma (St Louis, MO, USA). BAY87-2243 was purchased from Topscience (Shanghai, China).

### Plasmids and transfection

The full-length DNA fragments of SARS-CoV-2 genes were synthesized by Genscript (Nanjing, China). The genes were then subcloned and inserted into mammalian expression vectors, respectively, as indicated. Plasmid transfections were carried out with Lipofectamine 2000 (Invitrogen) according to the manufacturer’s instructions.

### Virus infection and sample collection

The virus was obtained from the Hubei Center of Disease Control, Wuhan, China, and then propagated in the Vero cell line. All experiments involving live SARS-CoV-2 infection were performed in a biosafety level 3 (BSL-3) laboratory in Hubei Center of Disease Control, Wuhan, China. After the virus infection, the samples were collected and inactivated, such as supernatant (56 °C, 45 min). RNA samples (Trizol Reagent treatment) and Western blotting samples (lysis buffer treatment and then add SDS loading buffer, at 100 °C for 15 min). After inactivation, all the samples were transferred to a biosafety level 2 (BSL-2) laboratory in Hubei Center of Disease Control, Wuhan, China, and carried out follow up experiments. Vesicular stomatitis virus (VSV, contains GFP) and herpes simplex virus-1 (HSV-1, contains GFP) strains were kindly provided by Dr. Bo Zhong of Wuhan University, Wuhan, China.

### Western blotting

The proteins were prepared and isolated by lysing the cells with lysis buffer (50 mM Tris-HCl, pH7.5, 0.5 mM EDTA, 150 mM NaCl, 1% NP40, and 1% SDS) containing the protease and phosphatase inhibitor cocktail with 1:100 dilution (Roche). The cell lysates were agitated at 4 °C for 1 h. The supernatants were then collected, boiled in the protein loading buffer for 5 min, and finally separated by SDS-PAGE. The treated samples were transferred onto the nitrocellulose membranes and then blocked with 5% milk in Tris-buffered saline with Tween-20 (TBST) for 1 h at room temperature (RT). The blots were probed at 4 °C overnight (O/N) with primary antibodies diluted in TBST containing 5% bovine serum albumin (BSA). The blots were washed two times for 10 min with TBST, probed with HRP-conjugated secondary antibody for 1 h, and then washed again. The blots were then imaged by using X-Ray film or under the LAS-4000 Imager.

### RT-PCR

The total RNAs from the tissues or cells were extracted with Trizol Reagent, which were then harvested with 0.25% trypsin EDTA (1×, Gibco Grand Island, NY, USA). The cDNAs were generated by the M-MVL reverse transcriptase. Specific primers and ChamQ SYBR qPCR Master Mix (Vazyme Biotech Co., Ltd, China) were used for the RT-PCR reactions. Each sample was tested in triplicate. Glyceraldehyde-3-phosphate dehydrogenase (GAPDH) was used as a normalization control. The conditions for PCR cycling were: 42 ^o^C for 5 min, 95 ^o^C for 10 s, and 40 cycles of 95 ^o^C for 5 s and 60 ^o^C for 30 s. All primers used were listed in Supplementary Table 6.

### ELISA

For enzyme-linked immunosorbent assay (ELISA), the ELISA Kits (BD Biosciences, San Jose, CA, USA) were used to determine the proteins expressed in the cell supernatants or in the sera according to the instructions provided by the manufacture.

### Cell viability

The cells were plated and grown in 96-well plates in triplicates. Cell proliferation and cell viability were assessed at different times after plating, as indicated, by using the Cell Counting Kit-8 (DOJINDO). Staining was performed according to the protocols provided by the manufacture

### Lentivirus

The construction of Lentivirus-Ctrl and Lentivirus-ORF3a and the production of virus for stable expression were provided by Addgene. Lentivirus constructs were transfected to the Phoenix packaging cell line. HEK293T cells were infected with the Lentiviral constructs and transfected with the packaging plasmids. The viral supernatants were filtered through a 0.45 μm filter, supplemented with 8 μg/ml polybrene, and then mixed with trypsinized recipient cells. Puromycin or hygromycin was used to select the infected cells.

### Immunofluorescence and confocal microscopy

The cells were cultured and grown in confocal culture dishes for 24 h and then fixed for 15 min with 4% paraformaldehyde. Then the cells were washed three times with PBS, permeabilized with 0.1% Triton X-100 for 15 min, washed again 3 times with PBS, and blocked in 5% BSA for 45 min. The cell dishes were incubated with 1% BSA or PBS containing primary antibodies at 4 ^o^C for 2 h or overnight, and then washed with PBS four times to remove unbound primary antibodies. The samples were incubated with the secondary antibody (FITC and Dylight-649) for 1 h and washed for three-time with PBS. Apply DAPI (1 µg/ml) for 10 min and washed thoroughly four times. The images were acquired on Olympus FV1000 Fluorescence Microscope. Mitochondrial membrane potential and ROS detection were determined by JC-1 Kit (Beyotime, C2006) or ROS Kit (Beyotime, S0033S). Staining was performed according to provided instructions.

### Flow cytometry

The JC-1 kit (Beyotime, C2006) or ROS kit (Beyotime, S0033S) was used for mitochondrial membrane potential and ROS detection. Staining was performed according to provided instructions. The cells were then measured by FCM (flow cytometry). At least 10,000 cells were counted and experiments were performed in triplicates.

### Statistical analysis

Data are means ± SD. Samples were compared via student’s *t*-tests using Prism 5 (GraphPad Software Inc.). *p* ≤ 0.05, significance threshold; ns, not significant (*p* > 0.05); *p < 0.05, **p < 0.01 and ***p < 0.001.

## Supplementary information

Supplementary Information

## Data Availability

All other data are included in the Article and Supplemental Information or available from the authors upon reasonable requests. Source data are provided with this paper.
