# Supplementary material for: HIF-1α promotes SARS-CoV-2 infection and aggravates inflammatory responses to COVID-19
Source: Signal Transduct Target Ther. 2021 Aug 18;6:308. doi: 10.1038/s41392-021-00726-w (PMC8371950; doi:10.1038/s41392-021-00726-w)
Supplement: Supplementary file 1 — Supplementary Information [file 41392_2021_726_MOESM1_ESM.pdf]

Supplementary Information

1. Supplementary Figures and Legends

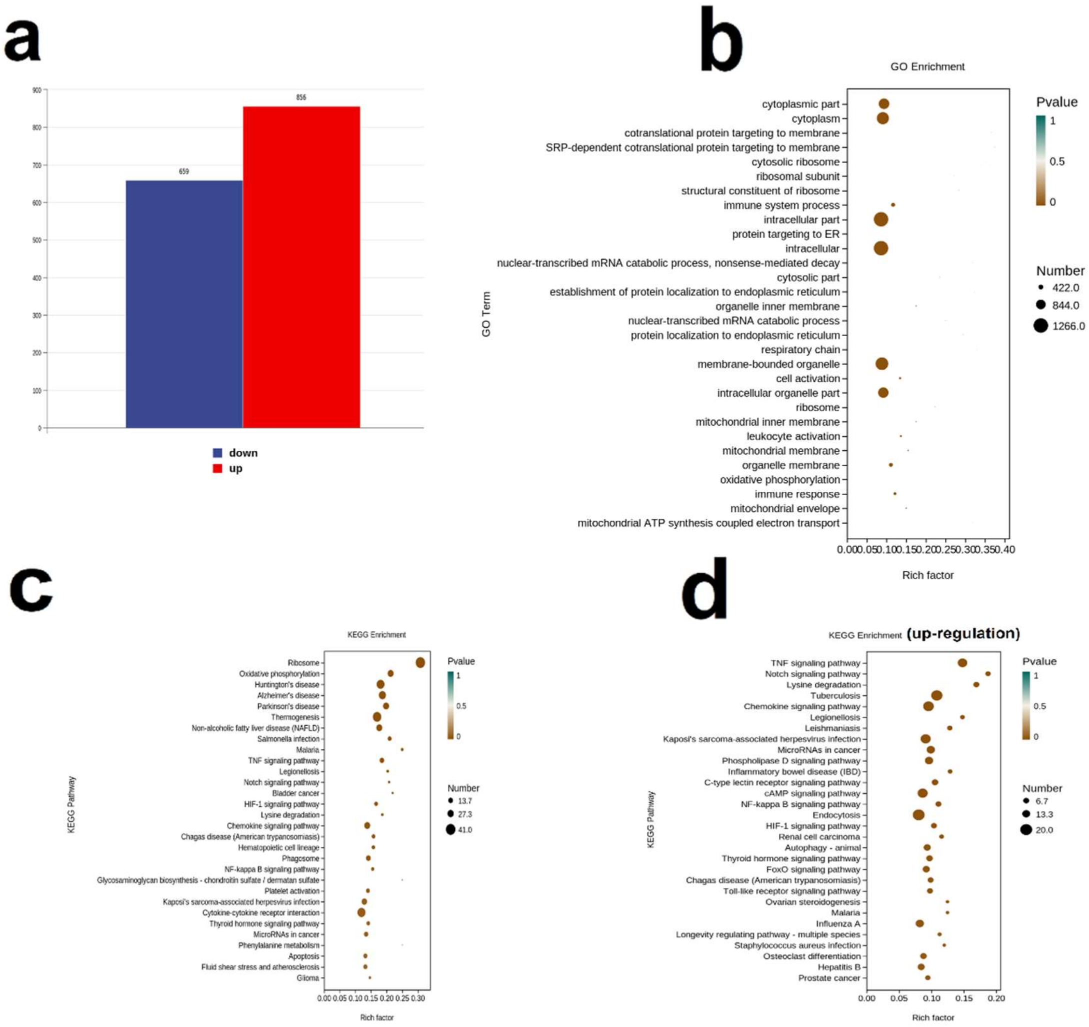

Supplementary Fig. 1. Immune and metabolism pathways are dysregulated in COVID-19 patients. (**a**) Up-regulated and down-regulated genes between COVID-19 patients and healthy individuals. (**b**) Point map of gene ontology enrichment of DEGs assigned to three classes as above. (**c**) Point map of the pathway enrichment (top 30) involved. (**d**) Point map of the up-regulated pathway enrichment (top 30) involved.

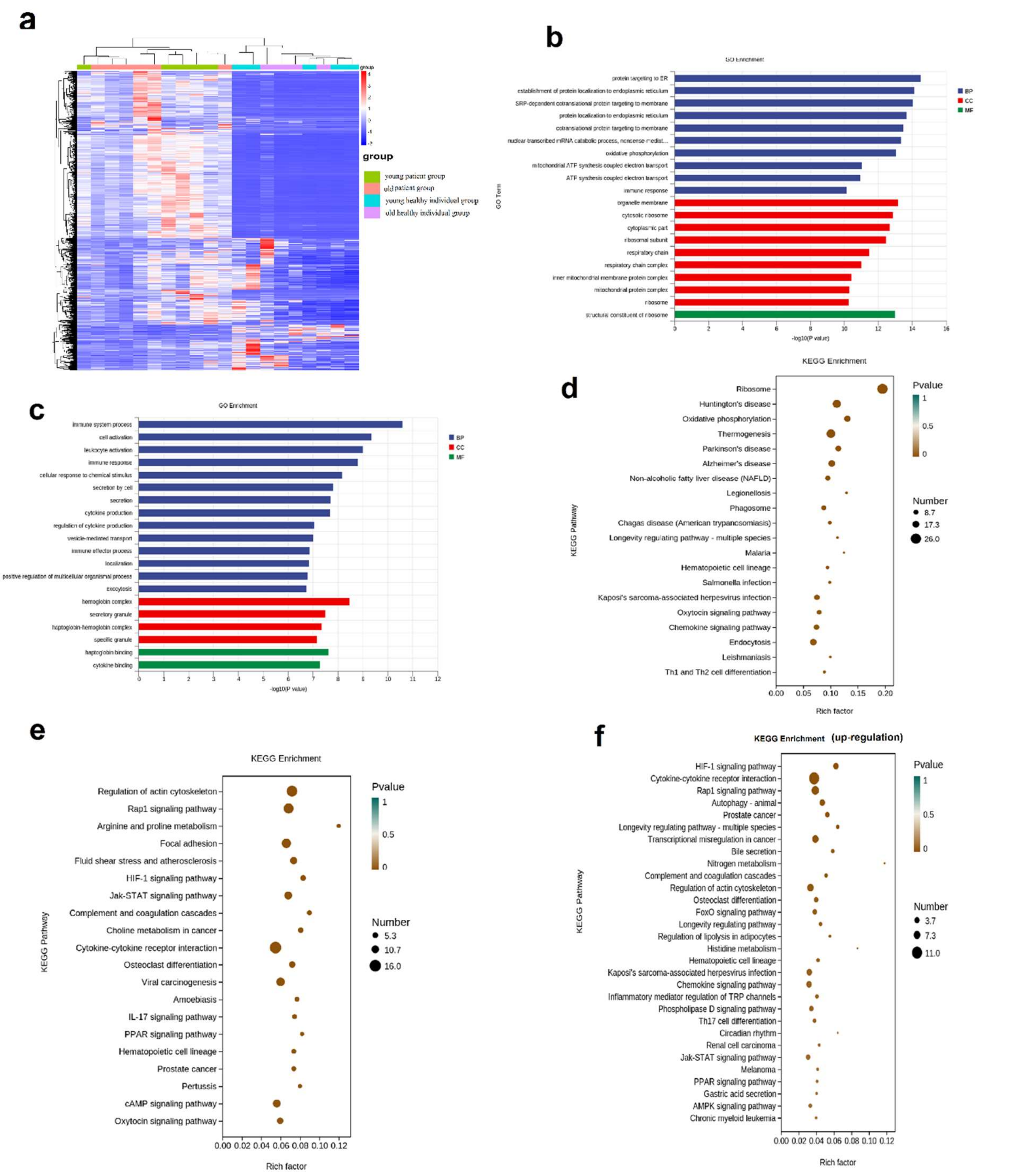

Supplementary Fig. 2. RNA sequencing (RNA-Seq) analyses of age dependent gene change.

(**a**) 11 COVID-19 patients and 9 healthy individuals divided to young healthy individual group, young patient group, elderly healthy individual group and elderly patient group (young: 35–50 years, old: 65–90 years). The differentially expressed genes are represented in scaled heatmap. (**b**) Histogram description of gene ontology enrichment of DEGs between young healthy individual

group and young patient group. (**c**) Histogram description of gene ontology enrichment of DEGs between elderly healthy individual group and elderly patient group. (**d**) List of the pathway enrichment (top 30) between young healthy individuals and young patients. (**e**) List of the pathway enrichment (top 30) between elderly healthy individuals and elderly patients. (**f**) List of up-regulated pathway enrichment between elderly healthy individuals and elderly patients.

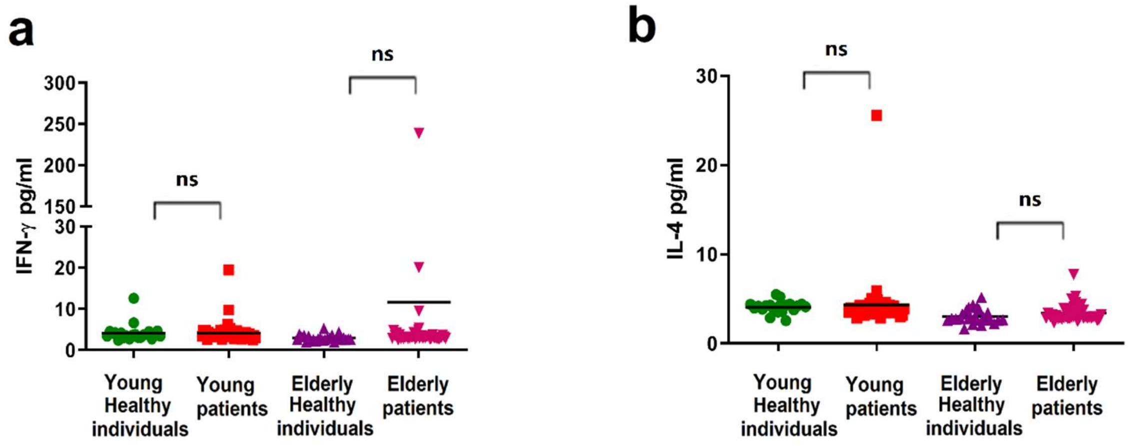

Supplementary Fig. 3. Elderly patients display excessive inflammatory responses and high mortality. (**a**, **b**) Blood samples were collected from healthy individuals (n=65) and COVID-19 patients (n=143) admitted to the Renmin Hospital, Wuhan University, Wuhan, China (Supplementary Table 3). 22 young healthy individuals, 49 young patients (20–50 years), 25 elderly healthy individuals, and 44 elderly patients (61–79 years) were sift out. IFN-γ protein (a) and IL-4 protein (b) expressed in the sera were determined by flow cytometry.

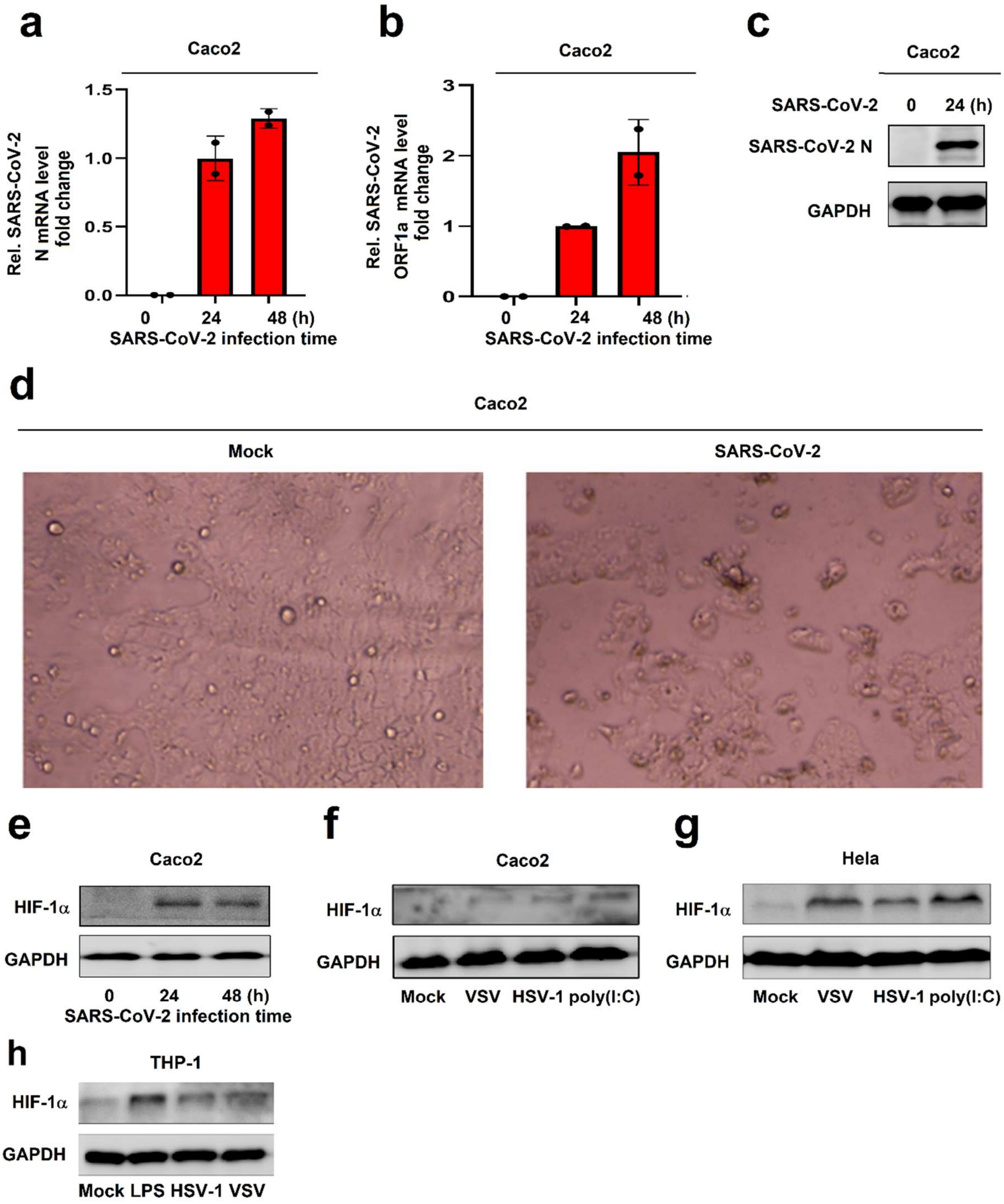

Supplementary Fig. 4. HIF-1α and immune-inflammatory cytokines are induced upon SARS-CoV-2 infection. (**a**, **b**) Caco2 cells were infection with SARS-CoV-2 for 24 and 48 h. The levels of SARS-CoV-2 N mRNA (a) and SARS-CoV-2 ORF1a (b) were determined by qRT-PCR. (**c**) Caco2 cells were infected with SARS-CoV-2 for 24 h. The levels of SARS-CoV-2 N

protein and GAPDH protein were analyzed by WB. (**d**) Caco2 cells were infected with SARS-CoV-2 for 48 h. Cell images showing the cell cytotoxicity. (**e**) Caco2 cells were infected with SARS-CoV-2 for 24 and 48 h. HIF-1α protein and GAPDH protein were determined by WB. (**f**) Caco2 cells were infected with VSV or HSV-1 or treated with poly(I:C). HIF-1α protein and GAPDH protein were determined by WB. (**g**) Hela cells were infected with VSV or HSV-1 or treated with poly(I:C). HIF-1α protein and GAPDH protein were determined by WB. (**h**) THP-1 cells were infected with VSV or HSV-1 for 24 h or treated with LPS (100 ng/ml) for 8 h. HIF-1α protein and GAPDH protein were determined by WB.

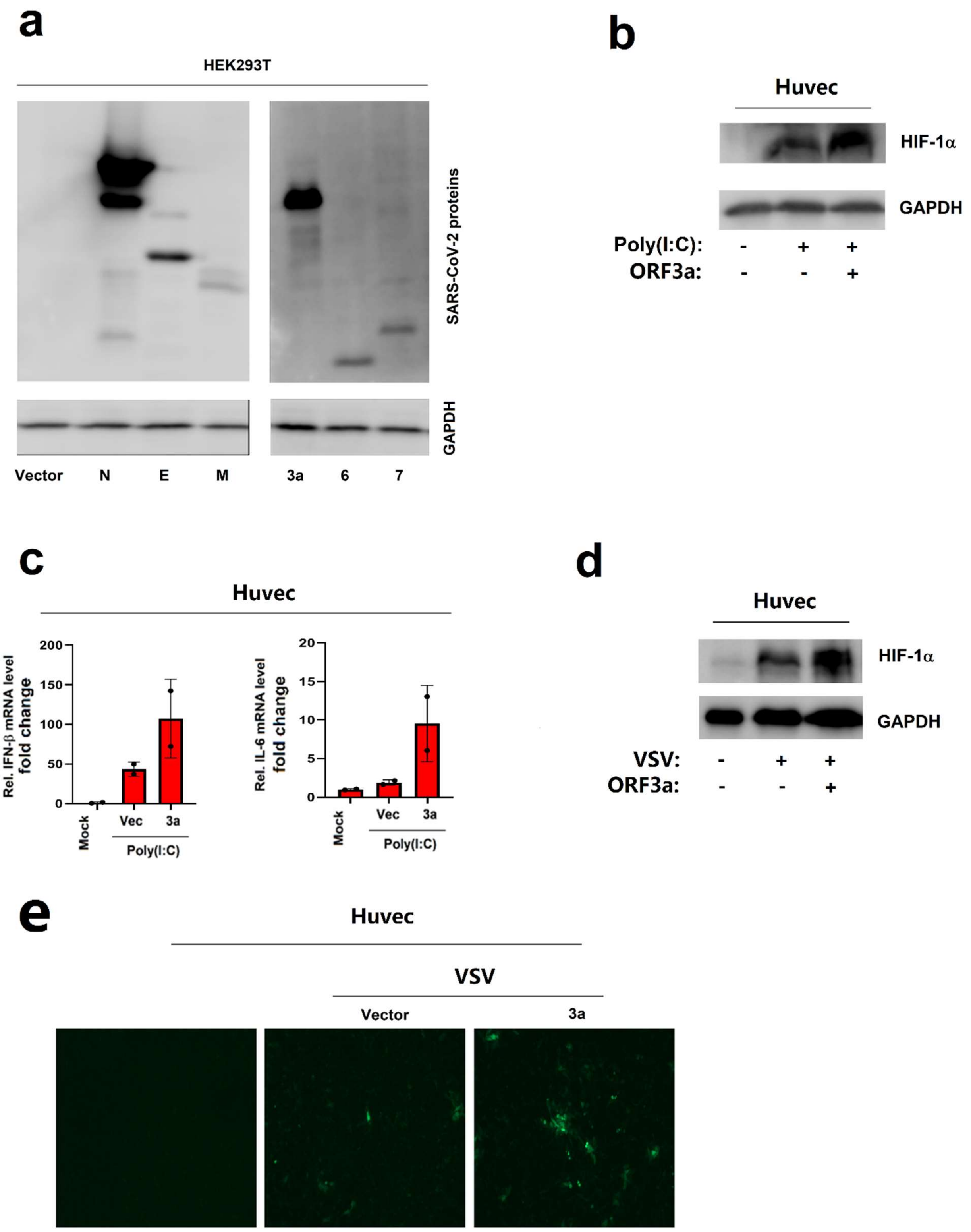

Supplementary Fig. 5. SARS-CoV-2 ORF3a promotes HIF-1α production and inflammatory responses. (**a**) Human HEK293T cells were transfected with indicated plasmids for 24 h. SARS-

CoV-2 proteins and GAPDH protein were assessed by WB. (**b**) Huvec cells were transfected with SARS-CoV-2 ORF3a for 24 h, treated with poly(I:C). HIF-1α protein and GAPDH protein were determined by WB. (**c**) Huvec cells were transfected with SARS-CoV-2 ORF3a for 24 h, treated with poly(I:C), IFN-β and IL-6 mRNAs were analyzed by RT-PCR. (**d**, **e**) Huvec cells were transfected with indicated plasmids for 24 h and then infected with VSV 24 h. HIF-1α and GAPDH proteins were assessed by WB (d) Cell images showing the virus infection (e).

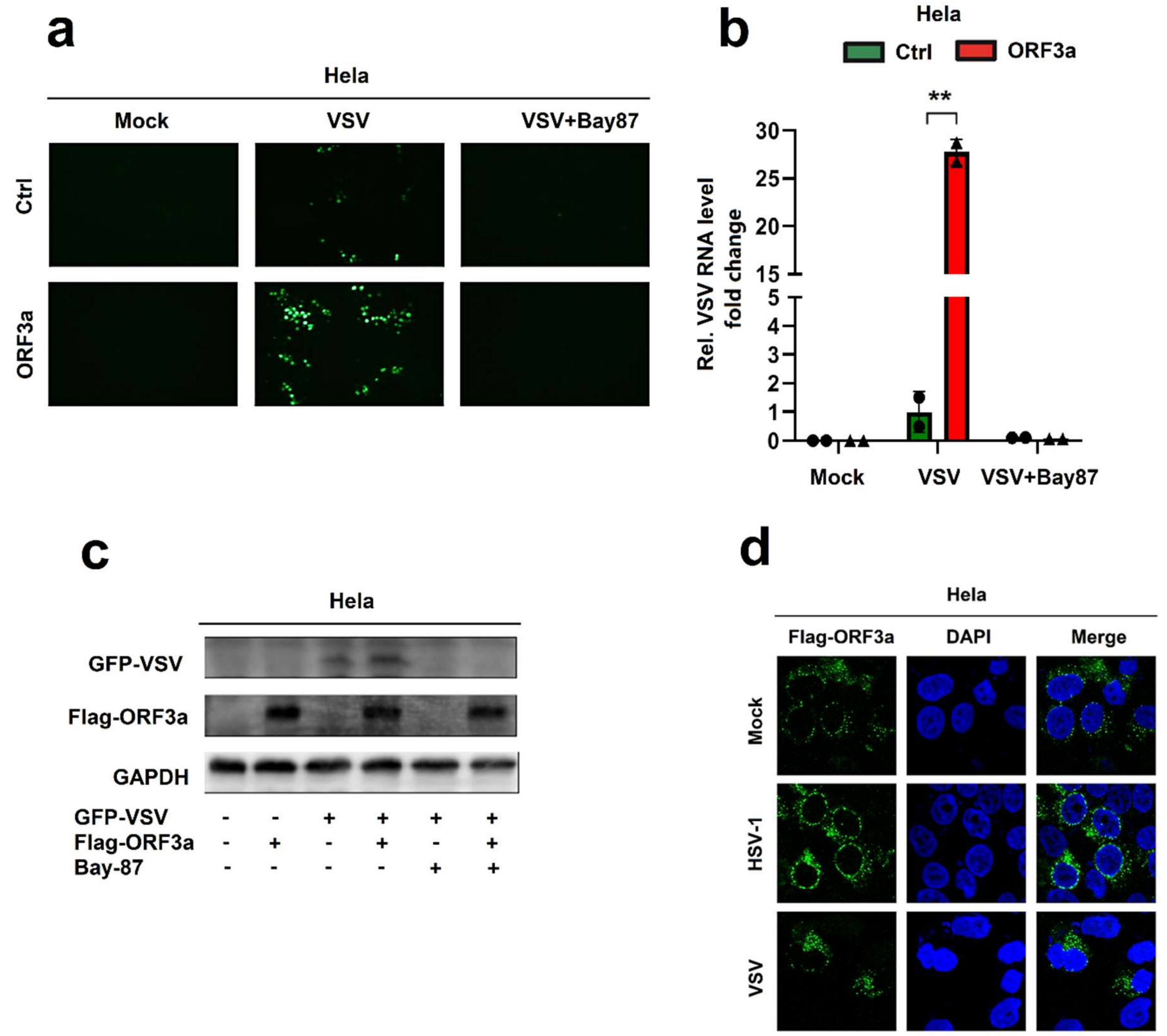

Supplementary Fig. 6. SARS-CoV-2 ORF3a promotes HIF-1α production and inflammatory responses. (**a**–**c**) Hela cells were transfected with indicated plasmids for 24 h and then infected with VSV 24 h. Cell images showing the virus infection (a). VSV mRNA was determined by RT-PCR (b). VSV-GFP and GAPDH proteins were assessed by WB (c). (**d**) Hela cells were transfected with indicated plasmids for 24 h and then infected with VSV or HSV-1 for 24 h. Protein locations were analyzed by confocal microscopy. DAPI were marker with nucleus.

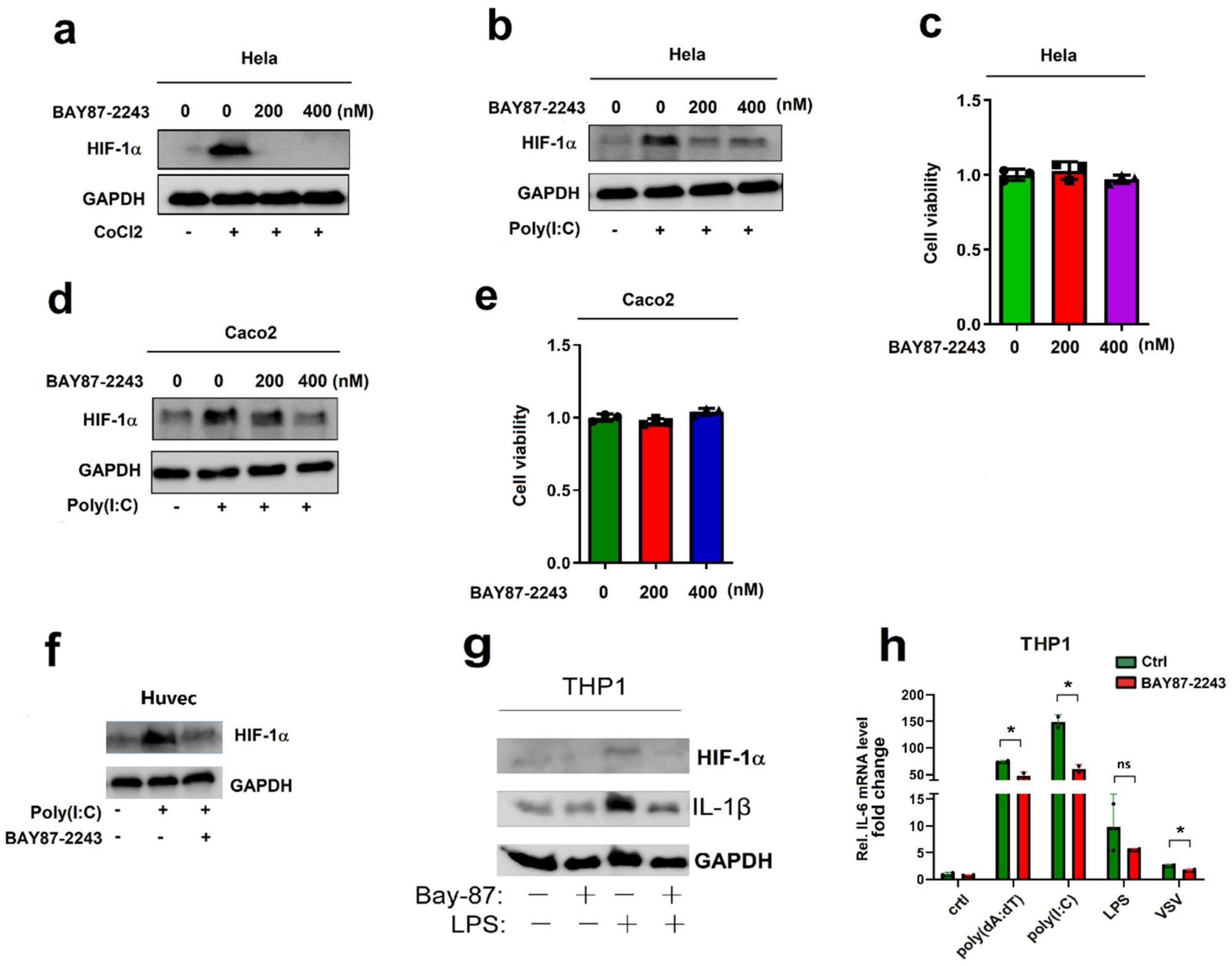

Supplementary Fig. 7. HIF-1α promotes SARS-CoV-2 infection and inflammatory responses.

(**a**) Hela cells were treated with $CoCl_2$ for 4 h, and then treated with BAY87-2243 for 6 h. HIF-1α protein and GAPDH protein were detected by WB. (**b**) Hela cells were treated with poly(I:C), and then treated with BAY87-2243. HIF-1α protein and GAPDH protein were detected by WB. (**c**) Hela cells were treated with BAY87-2243 at different concentrations. Cell viabilities were assessed by the CELL COUNTING Kit-8. (**d**) Caco2 cells were treated with poly(I:C), and then treated with BAY87-2243. HIF-1α protein and GAPDH protein were detected by WB. (**e**) Caco2 cells were treated with BAY87-2243. Cell viabilities were assessed by the CELL COUNTING kit-8. (**f**) Huvec cells were treated with BAY87-2243 and then treated with poly(I:C). HIF-1α and GAPDH proteins were determined by WB. (**g**) THP-1 cells were treated with BAY87-2243 , and then treated with

LPS. HIF-1α, IL-1β and GAPDH proteins were determined by WB. (**h**) THP-1 cells were treated with BAY87-2243 and then treated with indicated stimulator. IL-6 mRNAs were analyzed by RT-PCR.

2. Supplementary Tables

Supplementary Table 1. Information of patient group and healthy individual group used in RNA-seq analyses.

Group	Gender	n	Age (average)
Healthy individual	Male	5	61.2
	Female	4	
COVID-19 patient	Male	6	60.0
	Female	5	

Supplementary Table 2. Information of patients and healthy individuals with different age in RNA-seq analyses.

	Group	n
Healthy individual	Young healthy individual	5
	Old healthy individual	4
COVID-19 patient	Young patient group	5
	Old patient group	6

Supplementary Table 3. Information of patient group and healthy individual group.

Group	Gender	n	Age (average)
Healthy individual	Male	31	52.6
	Female	34	
COVID-19 patient	Male	67	56.4
	Female	76	

Supplementary Table 4. The age distribution of death patients (year).

**Under 50**	**51–64**	**65–80**	**Over 80**
13	59	115	35

Supplementary Table 5. Information of death patients.

Project	Number
**Total**	**222**
Male	151
Female	71
**Age (average)**	**68.8 years**
Cancer	8
Diabetes	44
Kidney diseases	4
Liver diseases	3
Cardiovascular diseases	37
**Death time (from morbidity to death)**	**20 days**

Supplementary Table 6. Primers used in this study.

Human IFN-β Forward:<br><br>5'-ATTGCCTCAAGGACAGGAG-3'	Human IFN-β Reverse:<br><br>5'-GGCCTTCAGGTAATGCAGAA-3'.
Human IL-1β Forward:<br><br>5'-CTCTCTCCTTTCAGGGCCAA-3'	Human IL-1β Reverse:<br><br>5'-GAGAGGCCTGGCTCAACAAA-3'
Human IL-6 Forward:<br><br>5'-AGACAGCCACTCACCTCTTCAG-3'	Human IL-6 Reverse:<br><br>5'-TTCTGCCAGTGCCTCTTTGCTG-3'
Human TNFα Forward:<br><br>5'-TCAGCCTCTTCTCCTTCCTG-3'	Human TNFα Reverse:<br><br>5'-TGAAGAGGACCTGGGAGTAG-3'
VSV Forward:<br><br>5'-ACGGCGTACTTCCAGATGG-3'	VSV Reverse:<br><br>5'-CTCGGTTCAAGATCCAGGT-3'
HSV-1 Forward:<br><br>5'-TGGGACACATGCCTTCTTGG-3'	HSV-1 Reverse:<br><br>5'-ACCCTTAGTCAGACTCTGTTACTTACCC-3'
Human HIF-1α Forward:<br><br>5'-CGTCGAAAAGAAAAGTCTCGAGAT-3'	Human HIF-1α Reverse:<br><br>5'-AGGCCTTATCAAGATGCGA ACT-3'
Human GAPDH Forward:<br><br>5'-CGGAGTCAACGGATTTGGTC-3'	Human GAPDH Reverse:<br><br>5'-GACAAGCTTCCCGTTCTCAG-3'
CoV-ORF1α Forward:<br><br>5'-CCCTGTGGGTTTTACACTTAA-3'	CoV-ORF1α Reverse:<br><br>5'-ACGATTGTGCATCAGCTGA-3'
CoV-N Forward:<br><br>5'-GGGGAACTTCTCCTGCTAGAAT-3'	CoV-N Reverse:<br><br>5'-CAGACATTTTGCTCTCAAGCTG-3'
